# Bibliography

**Published:** 1893-05

**Authors:** 


### Bibliography.

ORTHODONTIA. By Dr. S. H. Guilford. New edition. Philadelphia, 1893.

We have been favored with an examination of the advanced sheets of this edition, which will be completed and for sale by the S. S. White Dental Manufacturing Company before this notice reaches our readers.

The first edition was so favorably received that another has become necessary. This has been nearly all rewritten, and every chapter altered to bring the work up to the present standard. Forty-two pages have been added and twenty-seven cuts have been taken out and fifty-two new ones inserted, with two pages of plate illustrations. Two new chapters have been added,—one on "Construction of Regulating Appliances" and one on "Electro-Plating;" besides these, many important contributions giving value and interest to the book as a whole.

The work is a thoroughly practical one from the first chapter to the last, the author having condensed with a skilful hand, apparently making all his work subservient to the one idea of clearly stating processes, so that the beginner in this difficult branch might have no trouble in following modes described. The author, doubtless from his long experience as a teacher, has learned the valuable fact that conciseness and clearness of expression are essential factors in a text-book. It is certainly very satisfactory in this respect.

The chapter on "Construction of Regulating Appliances" is a very valuable and important addition. The author's practical knowledge and long experience have enabled him to give clear directions for the preparation of appliances and related mechanical work. His idea seems to be to make everything plain to the most uninstructed student, and that he has succeeded in this must be acknowledged.

This edition, with its many changes, may be regarded as a new book, as it brings up the subject to the latest date, comprising not only the author's work, but that of a large number of others engaged in the same line of labor.

For the practical man no better book can be at hand for continual reference.

General criticism may be indulged in on some points of practice. This is to be expected, for probably no branch of dentistry presents a greater variety of opinion as to the proper course to pursue in any given case. This variety of sentiment and practice has had a depressing effect on the untrained mind, inculcating the fear that the regulating of teeth is the one branch of dentistry to be dreaded and, if possible, shunned. It seems to the writer of this that Professor Guilford has made the path so easy that the very natural want of confidence will give way to a desire to test his methods, as well as those he quotes from other authors.

The illustrations are excellent throughout, rendering the text perfectly intelligible.

A Practical Treatise on Artificial Crown- and Bridge-Work. By George Evans. Third Edition, Revised and Enlarged, with six hundred and thirty-one illustrations. The S. S. White Dental Manufacturing Company, Philadelphia, 1893.

There is, perhaps, no better evidence of the advance made in mechanical dentistry than the introduction of such a work as this. Many of those yet in practice can well remember when but two forms of crowns were in use,—the old wooden pivot and the gold pin inserted in various ways, with tube and without. It is astonishing that these held so long in practice without any material change, but this came at last when other things of more importance had been, in a measure, settled, and the inventive mind was left free to turn its faculties in other directions.

From the time of the first introduction of other forms, a comparatively recent period, the growth in this direction has been the most remarkable of any of the procedures in dentistry. The truth of this is amply set forth in the very satisfactory volume just issued.

This, with its three hundred and forty-six pages and six hundred and thirty-one illustrations, sufficiently demonstrates the industry and care of the author to secure all improvements made in crown- and bridge-work, but so prolific has the labor been in this direction that many suggestions made since its issue must be deferred for a fourth edition.

After several chapters on the preparatory "Treatment of Teeth and Roots," the author begins with the consideration of the crowns, commencing with that of Bonwill, and then continues through the various forms of crowns up to the use of the "gold collar," and from this, by easy gradations, to bridge-work in all its different forms. So thoroughly is every step properly illustrated, that it would seem impossible for the beginner to fail in following the text and become an expert without any other aid to guide him.

The third edition seems to leave nothing to be desired as a text-book for students, and as a work of reference to those skilled in metal-work. In every respect this book is a credit to the author and the publishers, the S. S. White Dental Manufacturing Company. The illustrations are very superior in an artistic sense, and throughout there has been nothing omitted to make it satisfactory to the student and general reader.

